# Intradialytic complications among patients on twice-weekly maintenance hemodialysis: an experience from a hemodialysis center in Eritrea

**DOI:** 10.1186/s12882-020-01806-9

**Published:** 2020-05-05

**Authors:** Saud Mohammed Raja, Yemane Seyoum

**Affiliations:** Department of Internal Medicine, Orotta College of Medicine and Health Sciences and Orotta National Referral Hospital, Asmara, Eritrea

**Keywords:** ESRD, Twice-weekly, Hemodialysis, Complication, Developing country, Eritrea

## Abstract

**Background:**

Twice-weekly maintenance hemodialysis sessions in patients with end stage renal disease are commonly practiced due to economic constraints in developing countries including Eritrea. To ameliorate the paucity of data on the subject, our study aims to shed light on the patterns of intradialytic complications exclusively in patients undergoing twice-weekly hemodialysis in the country.

**Methods:**

A descriptive cross-sectional study was conducted from March 01 to July 31, 2018 at Dialysis Unit of Orotta National Referral Hospital, Asmara, Eritrea in patients with end stage renal disease undergoing twice-weekly hemodialysis. Hemodialysis sessions were assessed for intradialytic complications. Data were fed into and analyzed using Epi-Info and Microsoft Excel.

**Results:**

A total of 29 patients were included in the five-month study period. Males were 19 (65.5%) and females were 10 (34.5%). More than half of the patients had diabetes. Out of the total 573 hemodialysis sessions, 176 (30.7%) of them involved one or more intradialytic complication. Hypotension was the most common complication occurring in 10% of the sessions followed by nausea and vomiting (5.24%), hypertension (5.06%), muscle cramps (4.71%), and headache (4.54%). Other complications such as back pain, chest pain, fever, chills and itching occurred in less than 3% of the sessions. There was no death immediately associated with the complications. Half of the intradialytic complications occurred in patients with diabetes. There was a positive correlation between intradialytic hypotension and diabetes, ultrafiltration volume as well as eating during hemodialysis. Use of central line catheter as a vascular access was associated with higher complication rate.

**Conclusion:**

Twice-weekly hemodialysis for end stage renal disease patients probably has similar intradialytic complications as the “standard” thrice-weekly frequency. Although twice-weekly hemodialysis schedule is certainly unsuitable for some patients, its advantage of preserving residual kidney function can prevent excessive interdialytic weight gain and thus lowering the risk of intradialytic hypotension related with higher ultrafiltration rate. Being the first study in the country on dialysis complications, we recommend further large scale research in the future.

## Background

Hemodialysis is the most commonly utilized therapeutic intervention for patients with end stage renal disease (ESRD) [1]. Currently, the standard practice is intermittent in-center 3 to 5 hours of thrice-weekly hemodialysis (HD) in developed countries and many developing countries [2]. Due to economic challenges, however, twice-weekly HD is commonly practiced in several developing countries especially in Asia and Africa [3–5].

Although HD is generally a safe procedure, acute intradialytic complications are frequently encountered. The most commonly associated complications include hypotension, muscle cramps, nausea and vomiting, headache, pruritus, fever and chills. Many of the complications are associated with hypotension. Rarely, life-threatening complications such as arrhythmias and other cardiovascular complications occur [6].

Acute complications of HD are mostly reported in the literature with no clear distinction of hemodialysis frequency per week. Most HD patients in Eritrea receive twice-weekly sessions. No prior study on complications of HD was conducted in the country. Therefore, this study tries to shed light on the patterns and frequencies of intradialytic complications among patients undergoing twice-weekly HD in a resource-limited developing country such as Eritrea.

## Methods

A descriptive cross-sectional study was conducted from March 01 to July 31, 2018 at Dialysis Unit of Orotta National Referral Hospital, Asmara, Eritrea. The dialysis unit has a capacity of 8 regular dialysis machines with 7 working for regular maintenance dialysis of ESRD patients and a machine dedicated for acute or emergency dialysis. Two shifts of dialysis sessions are provided per day. With rare exceptions, the maintenance HD prescription in the dialysis unit is 4 hours of twice per week sessions for each patient.

All patients with end stage renal disease undergoing twice-weekly maintenance HD were enrolled into the study. Hemodialysis sessions in patients with acute kidney injury, undergoing emergency dialysis, ESRD patients on their initiation HD phase and patients on schedule other than twice-weekly were excluded from the study. A self-structured questionnaire for recording the HD prescription, vital signs and intradialytic complications was utilized for each patient in each session and filled carefully by the dialysis nurses. Intradialytic complications were listed in the questionnaire for checking them when they occur. Data collection was supervised by the dialysis physicians— the authors in this study.

Intradialytic hypotension (IDH) was defined as a fall of ≥20 mmHg in systolic blood pressure from the baseline or less than 90 mmHg systolic during the HD session with or without symptoms [7]. Intradialytic hypertension was defined as a rise in mean arterial pressure (MAP) > 15 mmHg within or immediately post dialysis [8]. Symptoms such as headache, backache, and itching were only included as intradialytic complications if the onset was during the HD session. Blood glucose for hypoglycemia was measured when considered clinically necessary. Electrocardiogram (ECG) was done for patients who complained of new-onset chest discomfort during HD.

Data were fed into a questionnaire designed in Epi-Info software version 7. Data cleaning and statistical analysis were conducted both in Epi-info 7 and Microsoft Excel 2007.

## Results

### Study population

A total of 29 patients were included in the five-month study period. Males were 19 (65.5%) and females were 10 (34.5%). The mean age was 53 with standard deviation of 16 and median age was 58 with a range from 22 to 72. All patients had negative serology for hepatitis B (HbSAg), hepatitis C and HIV with the exception of one with positive anti-HCV antibodies. More than half of the patients were diabetics. Table 1 shows the clinical causes of ESRD in the patients.

**Table 1:** Clinical causes of ESRD in the HD patients

Cause of ESRD	Frequency (*n* = 29)	Percentage
Diabetic nephropathy	14	48.3%
Chronic glomerulonephritis	4	13.8%
Unknown	4	13.8%
Polycystic kidney disease	2	6.9%
Diabetes and hypertension	2	6.9%
Hypertensive nephropathy	2	6.9%
Multiple myeloma	1	3.4%
Total	29	100.0%

The patients were on hemodialysis for a range of 4 months to 6 years. The dialysis vintage was less than 6 months in 2 (6.9%) patients, 6 months to less than a year in 13 (44.8%), a year to less than 2 years in 7 (24.1%), 2 to less than 3 years in 4 (13.8%), and 3 (10.3%) patients were on hemodialysis for more than 3 years. Apart from medications specific to the underlying cause, all patients were on calcium, vitamin D, and iron supplements unless contraindicated. Seven (24.1%) patients took erythropoietin alpha (Eprex®) injection during the study period. Fifteen (51.7%) patients were on oral diuretics (furosemide).

### Hemodialysis sessions

A total of 573 HD sessions were assessed. All study patients were in a twice-weekly HD schedule that is 2 to 4 hours per session. Hemodialysis duration was 4 hours in 312 (54.5%) sessions, 3 hours in 229 (40.0%) and 2 hours in 30 (5.2%) and 2.6 h in two (0.3%) of the 573 sessions. Standard low-flux membrane dialyzers and bicarbonate dialysate were used in all sessions. Anticoagulant utilized was unfractionated heparin. The mean blood flow was 294 ml/min with SD ± 21 and median 300 ml/min. The highest blood flow recorded was 350 ml/min. Dialysate flow was set at 500 ml/min in all sessions. Ultrafiltration (UF) was set based on an increase from target dry weight. The mean interdialytic weight gain was 2.1 kg (median 2 kg) with the maximum record being 5.5 kg. The mean UF volume was 2247 ml (median 2500 ml) and the maximum was 4000 ml per 4 hours session. The vascular access in almost half of the patients was arteriovenous (AV) fistula while in the other half being tunneled or temporary central lines as shown in Table 2.

**Table 2:** Complications and type of vascular access

Vascular access	No. of patients (%)	HD sessions	Sessions with Complications (%^a^)
AV Fistula	15 (51.7%)	384	81 (21.1%)
Temporary central line	8 (27.6%)	81	37 (45.7%)
Tunneled central line	6 (20.7%)	108	58 (53.7%)
Total	29 (100.0%)	573	176 (30.7%)

^a^% refers percentage out of the total HD sessions in those with a particular vascular access

### Intradialytic complications

**Fig. 1:**
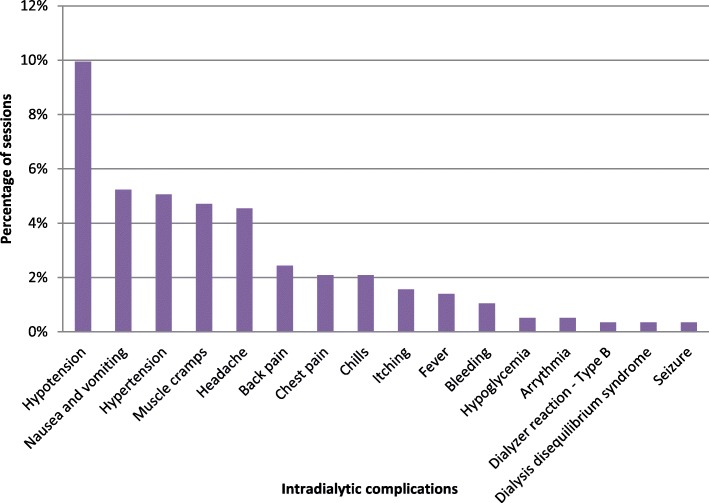
Fequencies of intradialytic complications

Out of the total 573 HD sessions analyzed, 176 (30.7%) involved one or more intradialytic complication. Hypotension was the most commonly encountered intradialytic complication occurring in nearly 10% of the sessions followed by nausea and vomiting (5.24%), hypertension (5.06%), muscle cramps (4.71%), and headache (4.54%) (Fig. 1). Other complications such as back pain, chest pain, fever, chills and itching occurred in less than 3% of the sessions. Half of the intradialytic complications occurred in patients with diabetes (Table 3). There were 3 hypoglycemic events, all of which in diabetic patients.

**Table 3:** Intradialytic complications stratified by diagnosis

Cause of ESRD	Frequency of complications	Percent
Diabetic nephropathy	88	50.0%
Unknown	34	19.3%
Chronic glomerulonephritis	26	14.8%
Diabetes & hypertension	9	5.1%
Polycystic kidney disease	8	4.5%
Hypertensive nephropathy	7	4.0%
Multiple myeloma	4	2.3%
Total	176	100.0%

**Fig. 2:**
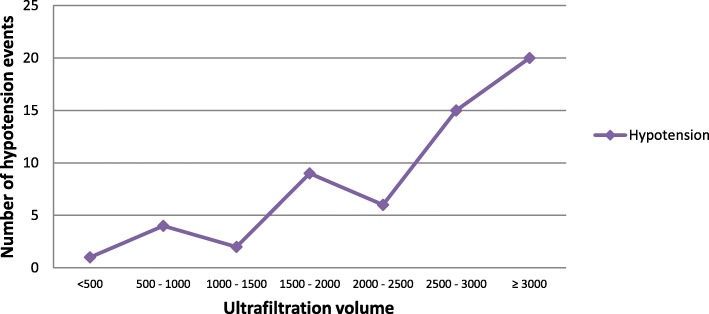
Trend of intradialytic hypotension with ultrafiltration volume

The cases of IDH were stratified by cause of ESRD and more than half of the cases were associated with diabetes (Table 4). Among the 57 cases of IDH, 49 (86%) occurred in those who took a meal prior to the session and 8 (14%) in those who came without eating just before the session. Eating during dialysis was also associated with 45 (79%) of IDH events. The instances of IDH increased with an increase in ultrafiltration volume per session as shown in Fig. 2. Out of the 30 events of nausea and vomiting, 13 (43%) were associated with hypotension.

**Table 4:** Intradialytic hypotension stratified by diagnosis

Cause of ESRD	Hypotension	Total sessions	% of the total hypotension
Diabetic nephropathy	32	325	56.1%
Chronic glomerulonephritis	11	70	19.3%
Diabetes and hypertension	4	24	7.0%
Multiple myeloma	4	6	7.0%
Hypertensive nephropathy	3	35	5.3%
Unknown	2	74	3.5%
Polycystic kidney disease	1	39	1.8%
Total	57	573	100.0%

The intradialytic complications were analyzed according to the type of vascular access of the patients. When seen as subgroups, the highest percentage of complicated sessions was in those with tunneled central line, where 58 (53.7%) out of 108 HD sessions were complicated (Table 2).

### Patients’ outcome

All the HD complications were successfully managed within the dialysis unit. There was no death immediately associated with intradialytic complication. Throughout the study period, 3 (10%) out of the 29 patients died. The causes of death were decompensated heart failure, decompensated chronic liver disease and severe septicemia. The causes of ESRD in two of them were diabetic nephropathy and one with hypertensive nephropathy. These 3 patients were undergoing HD using central line catheters — 2 with a temporary femoral line and 1 with a tunneled subclavian line.

## Discussion

The most common intradialytic complication is hypotension with an incidence ranging from < 5 to 40% of treatments depending on the definition of IDH [6]. There is no single standard definition for IDH [9]. The definitions range from a percentage decrease in systolic blood pressure to symptomatic hypotension requiring intervention. In our study, 10% of the sessions were complicated by hypotension in consistence with the bulk of the literature. A Chinese study shows that IDH was significantly lower in twice-weekly than thrice-weekly HD with 12.6% incidence in twice-weekly group compared to 27.5% in the thrice-weekly group with a *p-*value < 0.05 [10]. A plausible explanation is related to the preservation of residual kidney function (RKF) in twice-weekly schedule. The 10% IDH rate in our study approximates a lower range in comparison to the rates in the literature overall.

In our study, 16 (55%) out of the 29 patients were diabetics. Similarly, in a study done in a neighboring country, Ethiopia, 60.4% of ESRD patients on maintenance HD had prior history of diabetes [5]. Intradialytic hypotension is reported to be more common in diabetics than non-diabetic patients on maintenance HD [6, 11, 12]. More than half of the IDH events in our study occurred in diabetic patients. This indicates that diabetes is also an important risk factor for IDH in maintenance HD in our setting.

As shown in Fig. 2, there was a positive correlation between IDH and UF volume in our study. Almost one third of IDH events occurred in UF of 3000 ml and more and 60% in 2500 ml and more despite the mean UF being 2247 ml. Ultrafiltration volume is determined by interdialytic weight gain which was almost 2 kg on average. Theoretically, the longer interdialytic days in twice-weekly HD seem to lead to higher interdialytic weight gain and thus increased risk of hypotension due to more aggressive UF requirements. However, various studies show that the preservation of RKF in twice-weekly HD provides a better fluid control and no significant differences are reported in interdialytic weight gain between twice and thrice treatment regimens in groups with similar RKF [3, 13, 14]. However, patients with too much interdialytic weight gain and low RKF are not suitable for twice-weekly HD [2, 15]. Measurements of RKF were not available in our patients. Hence, the association of higher UF volume in many of IDH events in our patients could suggest that some of them might have been unsuitable for twice-weekly HD schedule due to either lower RKF or excessive interdialytic weight gain. Although UF volume but not UF rate was documented in our cases, a weight-based UF rate limit instead of fixed UF volume for interdialytic weight gain is suggested to decrease the incidence of IDH [13].

Regardless of the controversy whether the benefits of dietary restriction during hemodialysis outweigh the nutritional adverse effects, there is a well established evidence that eating just prior or during HD is a risk factor for IDH [16, 17]. Correspondingly, nearly 4 in 5 of the IDH events were associated with meal intake during HD sessions in this study.

The second most common complication was nausea and vomiting, many of which associated with IDH. Intradialytic hypertension is a common complication with a prevalence of 5 to 15% [8]. Five percent of HD sessions in our study were complicated by a significant rise in blood pressure during the session. Other common complications such as muscle cramps, headache, back pain, chest pain, itching, chills and fever were comparable with other similar studies [18–20], our rates being generally in the lower range. No rare fatal intradialytic complication occurred in this study.

We tried to observe the complications in relation to the vascular access of patients to verify whether those with central lines, who constituted nearly half of the cases, had added risks to intradialytic complications. Hemodialysis sessions in those with central lines as a vascular access had more complicated events than those with AV fistula. This finding could not be explained in this study or from the literature and further research is recommended. However, vascular access-related complications such as central line infections could also have a partial role.

This study is the first scientific documentation of intradialytic complications among ESRD patients on maintenance HD in Eritrea ever since the launch of dialysis service in the country. Being done in patients exclusively under twice-weekly HD sessions, our study has described the pattern of intradialytic complications in the setting to be comparable with reports from other dialysis centers in the region and worldwide.

Although our study provided important findings, there are some limitations worth mentioning. The study was a single center experience with a small population and relatively shorter period which could hinder making a robust inference. Residual kidney function was not measured as some variables for its calculation could not be fulfilled due to laboratory limitations. Body mass index (BMI) was also not calculated for the patients.

## Conclusion

Maintenance HD in ESRD, when conducted on twice-weekly schedule as in many developing countries, probably has similar intradialytic complications as the “standard” thrice-weekly HD frequency. Although twice-weekly HD is undoubtedly inappropriate for some patients, its advantage of preserving RKF can prevent excessive interdialytic weight gain and thus lowering the risk of IDH related with higher UF rate. In spite of being an initial informative study on the subject in Eritrea, we recommend a multi-center study to be conducted in the country that extends the knowledge not only on hemodialysis complications but also in all aspects of dialysis and kidney disease.

## Data Availability

The datasets used for this study are available from the corresponding author on reasonable request.

## Abbreviations

ESRD: End Stage Renal Disease
HD: Hemodialysis
UF: Ultrafiltration
AV: Arteriovenous
IDH: Intradialytic Hypotension
RKF: Residual Kidney Function
